# Ergodicity Breaking and Ageing in a Vibrational Motor

**DOI:** 10.3390/e27080802

**Published:** 2025-07-28

**Authors:** Yaqin Yang, Hongda Shi, Luchun Du, Wei Guo

**Affiliations:** 1 Key Laboratory of Artificial Microstructures in Yunnan Higher Education Institutions, School of Physical Science and Technology, Kunming University, Kunming 650214, China; 2 Department of Physics, Yunnan University, Kunming 650091, China

**Keywords:** ergodicity, ageing, vibrational motor

## Abstract

The ergodicity and ageing phenomena in a vibrational motor system driven by a periodic external force are investigated. Within the tailored parameter regime, the amplitude and frequency demonstrate contrasting effects on ergodicity. An increase of amplitude induces a transition from non-ergodic to ergodic behavior, whereas a higher driving frequency leads to a transition from ergodic to non-ergodic dynamics. These transitions are attributed to the enhanced ability of larger amplitudes to overcome potential energy barriers and the improved responsiveness of the system to external variations at lower frequencies. Moreover, pronounced ageing effects are observed at low amplitudes or high frequencies. These findings offer new insights into the intrinsic dynamical mechanisms of vibrational motor systems and provide a theoretical foundation for predicting their long-term operational performance.

## 1. Introduction

Ergodicity has always been a focal point of research in physics, chemistry, and biology, such as in quantum chaotic phenomena [1,2,3,4,5], continuous-time Markov processes [6], molecular collision and chemical reaction pathways [7,8,9], as well as molecular motion and diffusion in living cells [10,11]. According to Boltzmann–Gibbs statistics, if the observation time is much greater than the average waiting time, the system is ergodic [12]. However, conclusions drawn from single-molecule tracking experiments consistently contradict the assumption of ergodicity, and the phenomenon is referred to as ergodicity breaking [13,14]. The ergodicity breaking manifests in two different forms: (i) Strong ergodicity breaking, characterized by discontinuities in the state or phase space of the system, such as in the ageing of mean-field spin glasses [15] and quantum systems with local constraints [16]; (ii) Weak ergodicity breaking implies the existence of continuous regions, but the corresponding trajectories cannot completely sample them, even over an infinite period of time [17,18,19]. Experimentally, the ergodicity breaking has been observed in lipid particle diffusion within yeast cells [20], receptor motility in living cells [21], protein motility on human cell membranes [22], and single-particle tracking experiments [23]. Theoretically, the ergodicity breaking has been demonstrated in non-Hermitian many-body systems [24], constrained quantum systems [25], and Schwinger models [26].

The occurrence of ergodicity-breaking processes is typically accompanied by ageing [27,28,29]. Ageing refers to the dynamical process in which a system, remaining out of equilibrium over extended periods, exhibits observables (such as correlation and response functions) that explicitly depend on both the waiting time and observation time, thereby breaking time-translation invariance [27,30]. In disordered or glass systems, ageing manifests as slow relaxation dynamics, in which the ability of the system to explore its phase space is diminished, leading to an enhanced decomposition of ergodicity [31]. The link between ageing and ergodicity breaking has been demonstrated in physical systems ranging from spin glass to complex fluids, with ageing being the driving force behind the observed non-ergodic behavior [32,33].

Ergodicity is essential for understanding the behavior of a vibrational motor system over long time scales. As a periodic system operating under vibrational resonance conditions, the characteristic of the vibrational motor is that the traditional noise term is replaced by a time-periodic driving force with temporal symmetry [34,35,36]. In the prototype model of a vibrational motor [34], the introduction of additional driving terms can induce complex dynamic behaviors similar to randomness, which is a key aspect of understanding its dynamic response characteristics. By systematically modulating key parameters, the absolute negative mobility (ANM) phenomenon can be observed, with the system exhibiting a tendency to move in the direction opposite to the external force under vibrational driving [34]. At present, regarding the complex dynamical phenomena in the vibrational motor, existing studies have explored the control of anomalous transport by coexisting attractors [35], as well as anomalous diffusion and diffusion enhancement inside the vibrational motor [36]. However, although the dynamical phenomena in the vibrational motor have been extensively studied, research on the ergodicity and ageing characteristics of vibrational motors remains insufficient.

In this work, we investigate the ergodicity and ageing of a vibrational motor. We demonstrate that the emergence of non-ergodic behavior originates from two distinct mechanisms: at small amplitudes, the low-energy states of the system confine the particles within potential wells, whereas at high frequencies, the ability of the system to respond to external perturbations is limited, preventing adaptation to changes in external forces. Ageing is observed under both small amplitudes and high frequencies, indicating memory of the initial state and a waiting-time dependence of time-averaged observables. Additionally, we unveil the presence of anomalous diffusion in the system and elucidate the influence of external driving forces on the diffusion behavior.

The work is organized as follows: in Section 2 and Section 3 the model and observables are introduced, respectively. In Section 4 the theoretical analysis and numerical simulation of ergodicity properties are provided. Section 5 is dedicated to the discussion. And our conclusions are presented in Section 6.

## 2. Model

In the vibrational motor model, the dynamics of inertial particles moving in a spatially symmetric periodic potential under the influence of two time-periodic external forces is considered. The dimensionless equations of motion governing the particle dynamics are given as follows [34]:

(1) $$\ddot{x}+\gamma \dot{x}=-V^{\prime }\left(x\right)+a\cos\left(\omega t\right)+A\cos\left(\Omega t\right)+f,$$

where x=x(t) denotes the displacement of the inertial particle at time *t*, and γ is the friction coefficient. The dot and prime denote differentiation with respect to *t* and *x*, respectively. The external spatially symmetric periodic potential is V(x)=sin(2πx) with unit period. acos(ωt) represents the first time-periodic signal with frequency ω and amplitude *a*. Acos(Ωt) represents the second time-periodic signal with frequency Ω and amplitude *A*. The constant bias force *f* is chosen to be negative, zero, or positive.

The first time-periodic signal is strong and can throw the system out of equilibrium. In contrast, the second time-periodic signal is weak and produces effects similar to noise. In the absence of the second signal, the system exhibits remarkably rich dynamical behavior in the asymptotic long time limit, including periodic motion, quasi-periodicity, and chaos [37,38,39]. Upon the introduction of a second signal, a series of anomalous transport phenomena can be observed, by tuning its driving amplitude and frequency, such as absolute negative mobility and diffusion enhancement [34,36]. The friction coefficient γ characterizes the damping effect arising from the interaction between the particle and its surrounding environment [40]. In the vibrational motor system, friction plays a crucial role in determining the dynamical response and energy dissipation properties. The coefficient γ governs the balance between the driving forces and dissipative effects, thereby influencing the ability of the system to sustain persistent motion and efficiently explore the phase space [29,35,36]. In the overdamped regime (large γ), inertial effects vanish, and the particle motion becomes increasingly constrained, leading to enhanced localization [41]. In contrast, in the underdamped regime (small γ), inertial effects become prominent, giving rise to richer dynamical behaviors, such as diffusion enhancement and complex transport phenomena [29,34,36].

## 3. Observables

The ensemble-averaged mean squared displacement (MSD) for a wide variety of systems often follows a power law scaling with time over broad time scales. The MSD/2t can be defined as

(2) $$\frac{\langle x^{2}\left(t\right)\rangle -{\langle x\left(t\right)\rangle }^{2}}{2t}\propto t^{\alpha }.$$

Deviations from normal Brownian motion (α=0) are a hallmark of anomalous diffusion, which encompasses subdiffusion (α<0) and superdiffusion (α>0) [13,17,42,43,44]. Specially, α=1 and α= −1 respectively correspond to ballistic diffusion and confined state [13,42,43,44]. Subdiffusion typically arises in complex media where particle motion is constrained, such as the cytoplasm or glassy materials [45,46]. Superdiffusion can be induced by persistent driving forces or long-range correlation mechanisms, such as in ordered driven systems or active matter [47,48].

To further investigate the ergodic properties of the system, the time-averaged mean squared displacement (TMSD) is introduced, defined based on a single-particle trajectory as

(3) $$\bar{\delta^{2}\left(t\right)}=\frac{1}{t_{\omega }-t}\int_{0}^{t_{\omega }-t}{\left[x\left(t^{\prime }+t\right)-x\left(t^{\prime }\right)\right]}^{2}dt^{\prime },$$

where $t_{\omega }$ denotes the length of the time series and *t* denotes the lag time. The TMSD characterizes the spatial exploration capability of a particle along a single trajectory. If the distribution of TMSDs over multiple particles is sufficiently concentrated, the TMSD and MSD tend to coincide, indicating that the system is ergodic, i.e., time averages are equivalent to ensemble averages. In contrast, if TMSDs from different trajectories still exhibit significant variability, the time- and ensemble-averaged mean squared displacement (ETMSD) is introduced to further characterize the system

(4) $$\langle \bar{\delta^{2}\left(t\right)}\rangle =\frac{1}{N}\sum_{i=1}^{N}\delta_{i}^{2}\left(t\right).$$

The ETMSD characterizes the overall average diffusive behavior of the system, reflecting its spatial sampling capability across multiple trajectories and time intervals.

To quantify the degree of fluctuations in the TMSD across different trajectories and to further assess the ergodic properties of the system, the ergodicity-breaking (EB) parameter is introduced

(5) $$EB=\frac{\langle {\bar{\delta^{2}\left(t\right)}}^{2}\rangle -{\langle \bar{\delta^{2}\left(t\right)}\rangle }^{2}}{{\langle \bar{\delta^{2}\left(t\right)}\rangle }^{2}},$$

which provides an appropriate measure for describing the magnitude of fluctuations of TMSDs. When EB⟶0, it indicates that the diffusive behavior among different particle trajectories is consistent, and the system satisfies ergodicity. In contrast, EB≠0 implies the presence of ergodicity breaking, meaning that even over an infinitely long observation time, a single trajectory is unable to fully explore the accessible phase space [23,49,50]. Therefore, the EB parameter not only quantifies the fluctuation of time-averaged observables among individual trajectories, but also serves to characterize the capability of the system to sample the phase space [13]. For a stationary process, the vanishing of the EB parameter is a sufficient condition for ergodicity.Therefore, before using EB, the stationarity of the process must be tested or the ensemble and time average must be compared (our system is stationary for a long time) [17,51]. The ratio of the time and ensemble-averaged MSDs can be employed.

Further, the amplitude scatter distribution Φ(ξ) of individual TMSD of trajectories is considered. The dimensionless parameter ξ can be defined as

(6) $$\xi =\frac{\bar{\delta^{2}\left(t\right)}}{\langle \bar{\delta^{2}\left(t\right)}\rangle }.$$

According to the Boltzmann–Khinchin ergodic hypothesis, the values ξ=1 and ξ≠1 indicate, respectively, that the system does and does not satisfy ergodicity [17,52,53]. Additionally, physical observables, such as ETMSD, may potentially depend on the time interval $t_{a}$ between the system’s initialization and the commencement of the measurement

(7) $$\langle \bar{\delta^{2}\left(t;t_{a}\right)}\rangle =\frac{1}{t_{\omega }-t}\int_{t_{a}}^{t_{a}+t_{\omega }-t}\langle {\left[x\left(t^{\prime }+t\right)-x\left(t^{\prime }\right)\right]}^{2}\rangle dt^{\prime },$$

where $t_{a}$ is the ageing time. An ageing factor can be defined as

(8) $$\Lambda =\frac{\langle \bar{\delta^{2}\left(t;t_{a}\right)}\rangle }{\langle \bar{\delta^{2}\left(t;0\right)}\rangle },$$

and Λ=1 indicates that there is no ageing in the system [54].

## 4. Results

**Figure 1:**
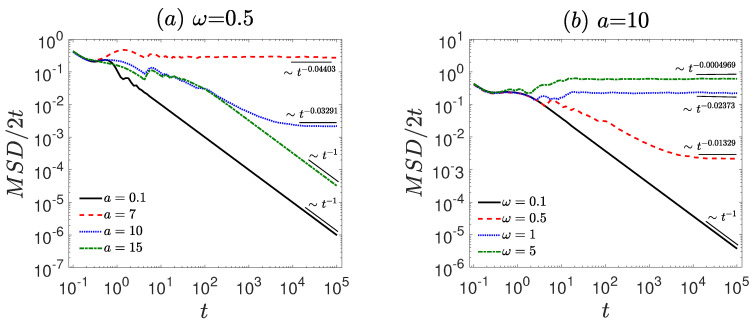
The MSD/2t is governed by the dynamical Equation (1). (**a**) Different amplitudes *a* = 0.1, 7, 10, 15 with fixed frequency ω = 0.5. (**b**) Different frequencies ω = 0.1, 0.5, 1, 5 with fixed amplitude *a* = 10. The diffusion exponent is extracted by power-law fitting (black thin lines). The remaining parameters are set as γ=1.35, A=0.01, Ω=0.05, and f=0.2. The diffusion behavior evolves with variations in amplitude and frequency, as indicated by the fitted black thin lines.

**Figure 2:**
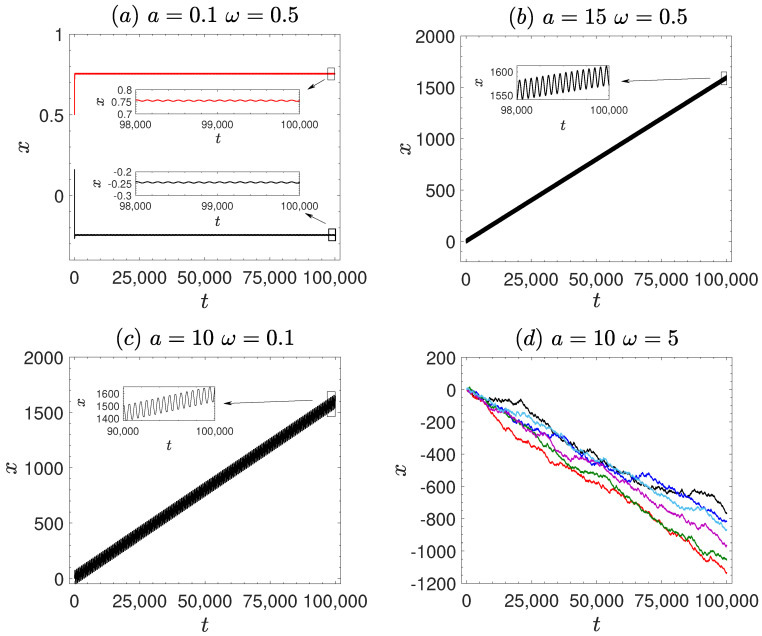
Typical trajectories in the vibrational motor correspond to Figure 1, with each color representing a different initial condition. (**a**) ω=0.5, a=0.1. (**b**) ω=0.5, a=15. (**c**) ω=0.1, a=10. (**d**) ω=5, a=10. The remaining other parameters are the same as Figure 1.

**Figure 3:**
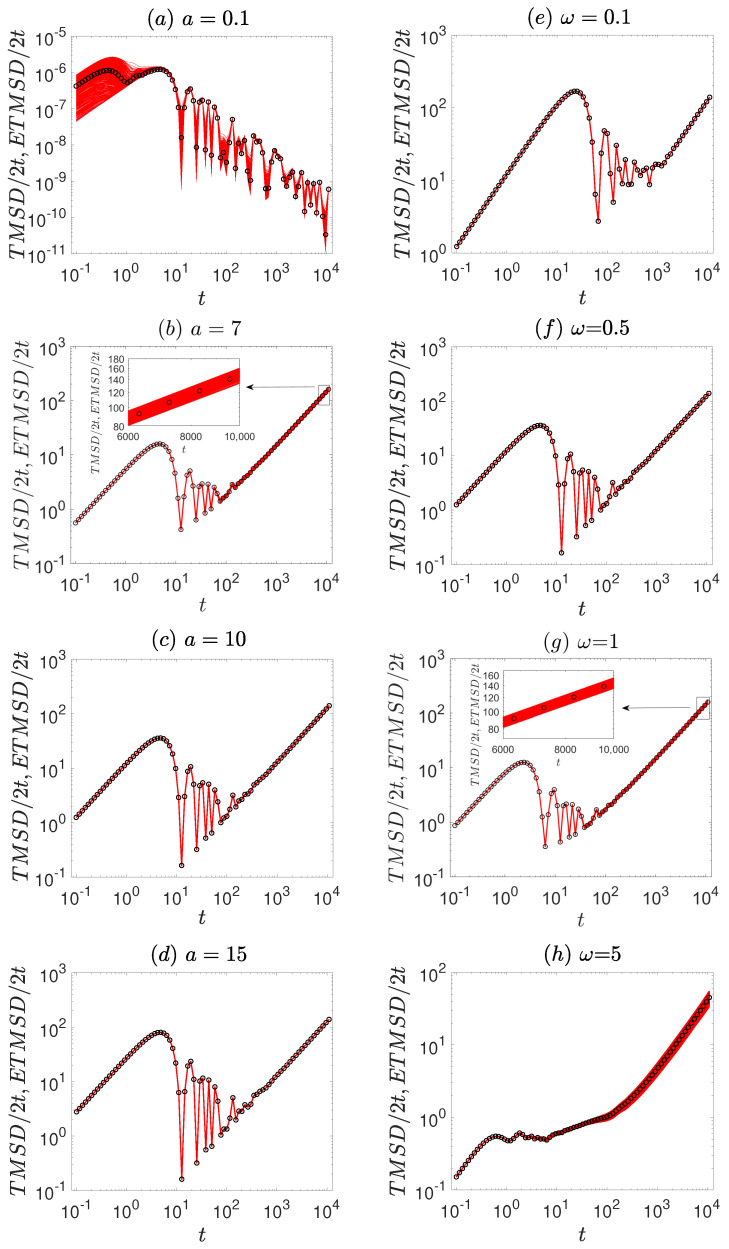
The TMSD/2t (marked by red solid lines) and ETMSD/2t (marked by black symbols) are governed by the dynamic Equation (1). The left column (**a**–**d**) corresponds to different external force amplitudes (*a* = 0.1, 7, 10, 15, respectively) with a fixed frequency ω=0.5. The right column (**e**–**h**) corresponds to different driving frequencies (ω = 0.1, 0.5, 1, 5, respectively) with a fixed amplitude *a* = 10. The insets in panels (**b**,**g**) are enlarged to provide a more detailed view over specific time intervals. The remaining other parameters are the same as Figure 1.

**Figure 4:**
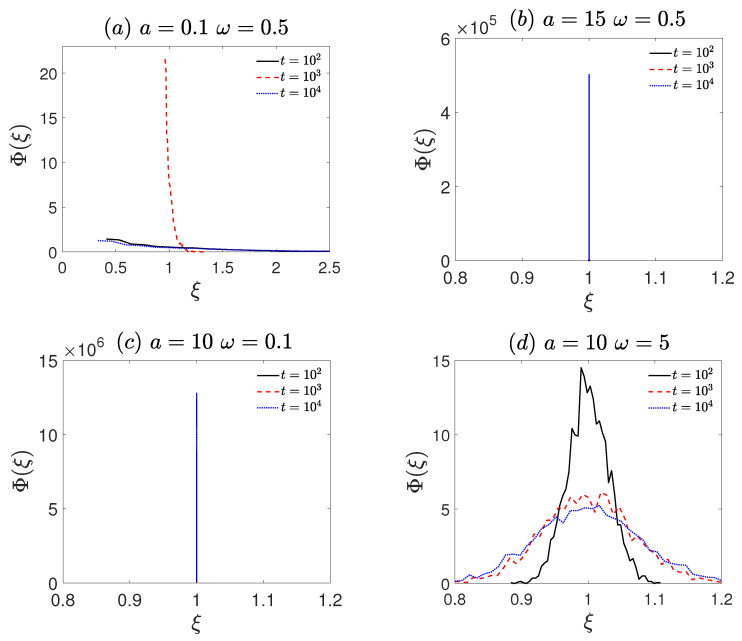
The amplitude scattering distribution ($\Phi \left(\xi \right)$, Equation (6)) corresponds to Figure 2. (**a**) The frequency ω = 0.5 and amplitude *a* = 0.1. (**b**) The frequency ω = 0.5 and amplitude *a* = 15. (**c**) The frequency ω = 0.1 and amplitude *a* = 10. (**d**) The frequency ω = 5 and amplitude *a* = 10. The remaining other parameters are the same as Figure 1.

**Figure 5:**
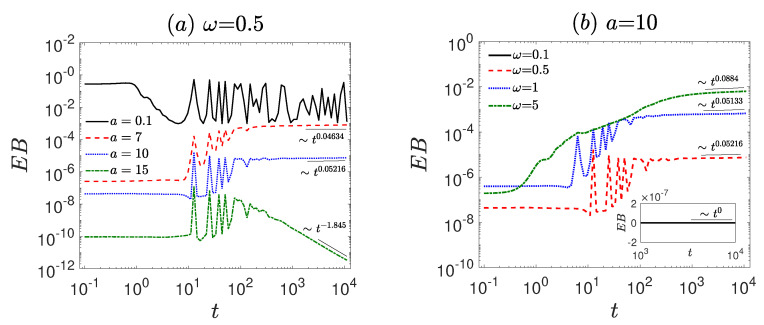
The ergodicity-breaking parameter (EB, Equation (5)) corresponds to Figure 1. (**a**) The different external force amplitudes with a fixed frequency ω=0.5. (**b**) The different driving frequencies with a fixed amplitude *a* = 10. The remaining other parameters are the same as Figure 1.

**Figure 6:**
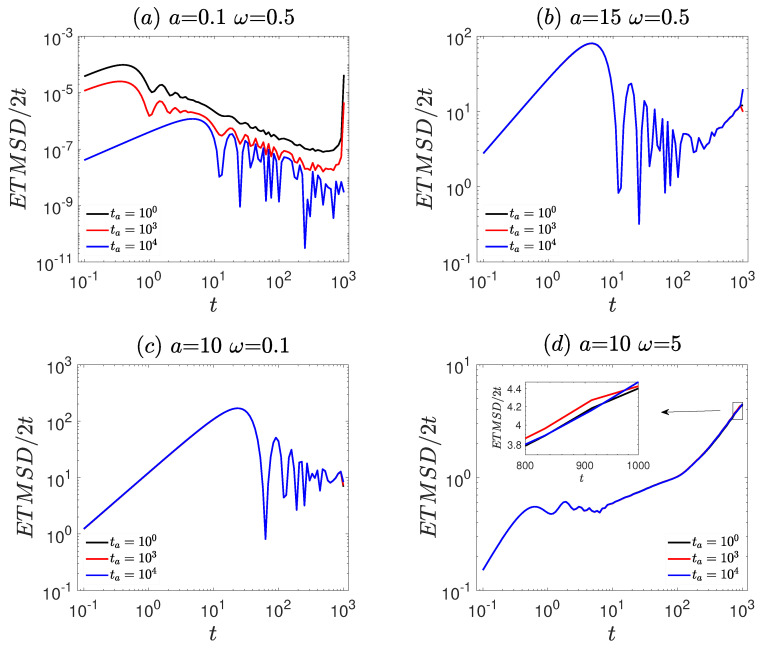
The ageing characteristics of the vibrational motor are governed by the dynamic Equation (7). (**a**) The frequency ω = 0.5 and amplitude *a* = 0.1. (**b**) The frequency ω = 0.5 and amplitude *a* = 15. (**c**) The frequency ω = 0.1 and amplitude *a* = 10. (**d**) The frequency ω = 5 and amplitude *a* = 10. The remaining other parameters are the same as Figure 1.

**Figure 7:**
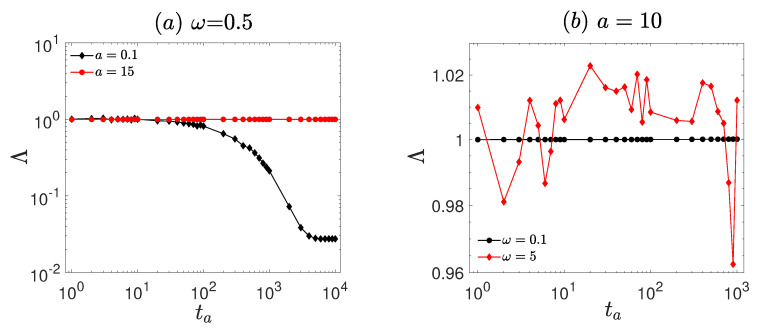
The ageing factor (Λ, Equation (8)) corresponds to Figure 6. (**a**) different amplitudes *a* (with fixed frequency ω=0.5). (**b**) different driving frequencies ω (with fixed amplitude a=10). The remaining parameters are the same as in Figure 1.

The numerical simulations of Equation (1) are performed using the second-order Runge–Kutta method. The time step is set to Δt=0.001, and the ensemble average is taken over N=3000 trajectories, with uniformly distributed initial conditions. To investigate the ergodicity and ageing properties of the system, we consider the amplitude *a* and frequency ω of the first time-periodic signal as control parameters. First, we analyze the behavior of MSD/2t for various values of *a* and ω, as shown in Figure 1, discussing the diffusion characteristics of the system. In Figure 2, we present the representative particle trajectories under different parameter sets to provide intuitive insight into the dynamical behavior of the system. Subsequently, we examine TMSD/2t and ETMSD/2t, illustrated in Figure 3, to explore the ergodicity of the system. The amplitude scattering function (Φ(ξ), Figure 4) and ergodicity-breaking parameter (EB, Figure 5) are examined to further assess the ergodic properties of the system. Finally, the ageing behavior of the system and relevant ageing factors are analyzed (Figure 6 and Figure 7).

Firstly, the anomalous diffusion behavior in the vibrational motor system is investigated (see Figure 1). For smaller amplitudes (a=0.1, see Figure 1a), the system is confined (α≈ −1). Under moderate external forces (e.g., a=7 and a=10), the system exhibits normal diffusion (α≈0). For relatively large amplitudes (e.g., a=15), the motion becomes confined again. As the amplitude increases, the system transitions from a confined state to normal diffusion, and at even higher amplitudes becomes confined again due to strong external driving.

Compared to amplitude, the influence of driving frequency on diffusion behavior exhibits a significantly different trend at lower driving frequencies. For a small driving frequency (ω=0.1, see Figure 1b), the system exhibits confined behavior characterized by α≈ −1. However, for moderate or large driving frequencies(ω=0.5, 1, and 5), the system displays normal diffusion (α≈0). As the amplitude of the external force increases, the diffusion changes from a confined state to normal diffusion.

To further understand the dynamical behavior of the particle, Figure 2 presents representative trajectories of a single particle under different parameter conditions. In Figure 2a, for a small amplitude *a* = 0.1, the particle remains confined near the symmetric periodic potential wells and only exhibits minor local oscillations. In contrast, Figure 2b corresponds to a large amplitude *a* = 15, where the driving force is significantly enhanced, allowing the particle to overcome the potential barriers and display a clear linear drift. In Figure 2c, under a low driving frequency ω = 0.1, the particle also exhibits pronounced directed motion, accompanied by strong periodic modulation. By comparison, Figure 2d shows the case of a higher frequency ω = 5, where the particle motion becomes more random and diffusive, and the trajectory overall displays a trend of gradual decay.

Meanwhile, in Figure 3, the relationship between TMSD/2t and ETMSD/2t is considered. For smaller amplitudes, the TMSD/2t is scattered around the ETMSD/2t in Figure 3a,b. This means that the system is non-ergodic. For larger amplitudes, the TMSD/2t curves in Figure 3c,d are close to each other. This indicates that the system regains its ergodicity. Therefore, as the amplitude increases, the system changes from the ergodicity breaking to ergodicity. However, the effect of driving frequency on ergodicity is the opposite of the effect of amplitude on the ergodicity of the system. With the increase of driving frequency, the system changes from ergodicity to ergodicity-breaking (Figure 3e–h).

To elucidate the above non-ergodicity properties, the amplitude scattering distribution Φ(ξ) is employed to analyze the ergodicity of the system. At long time scales (especially at $t=10^{4}$), the distribution of ξ is shown in Figure 4a–d. For a small amplitude (a=0.1 and ω=0.5, Figure 4a), Φ(ξ) exhibits a broad, right-skewed profile, with the probability density primarily distributed at ξ<1, indicating non-ergodicity. For a large amplitude (a=15 and ω=0.5, Figure 4b), Φ(ξ) exhibits a pronounced peak at ξ=1, i.e., ergodicity. For low frequency (a=10 and ω=0.1, Figure 4c), a pronounced peak at ξ=1 is also observed, indicating ergodicity. In contrast, for high frequency (a=10 and ω=5, Figure 4d), the distribution becomes substantially broader and more dispersed, with the probability density at ξ=1 markedly reduced compared to the ergodic cases, signifying ergodicity breaking. Overall, the amplitude scattering distribution further confirms the non-ergodic characteristics observed in Figure 3.

In addition, the ergodicity of the system is quantitatively analyzed through the ergodicity-breaking parameter (EB). For small amplitudes (see Figure 5a), the system is ergodicity-breaking (EB≠0). As the amplitude increases, the EB value decreases (EB≈0). This means that as the amplitude increases, the system changes from non-ergodicity to ergodicity. However, the amplitude and frequency demonstrate contrasting effects on ergodicity. As frequency increases, the system changes from ergodicity to non-ergodicity (Figure 5b).

The ageing characteristics of the system are presented in Figure 6. As observable from Figure 6, the behavior of ETMSD/2t exhibits a clear dependence on the ageing time $t_{a}$. Specifically, Figure 6a,d shows clear trends, indicating significant ageing characteristics. However, in Figure 6b,c, the effect of ageing time on ETMSD/2t is relatively small, indicating that the ageing characteristics of the system are not significant. These observations demonstrate that the ageing effect is enhanced under small amplitudes or high driving frequencies.

The ageing behavior of the system is characterized by the ageing factor Λ, as shown in Figure 7. At a fixed frequency (ω=0.5, Figure 7a), Λ remains constant at unity (Λ=1) for large amplitude (a=15). In contrast, for small amplitude (a=0.1), Λ decreases significantly as the ageing time $t_{a}$ increases and eventually stabilizes at Λ=0.02, indicating pronounced ageing effects. At a fixed amplitude (a=10, Figure 7b), Λ is stable at unity (Λ=1) at low frequency (ω=0.1). For high frequency (ω=5), significant fluctuations of Λ around unity are observed, indicating the presence of ageing.

## 5. Discussion

We investigate the influence of amplitude and frequency with respect to the ergodicity and ageing of a vibrational motor system. Regarding the ergodicity of the vibrational motor, an increase of amplitude induces a change from non-ergodic to ergodic behavior (Figure 3a–d). At smaller amplitudes, the system possesses lower energy and becomes localized within potential wells. This confinement hinders the ability of the system to effectively explore the entire phase space, leading to non-ergodic behavior. As observed in disordered systems, changes in energy states significantly impact ergodicity [27,28]. In our vibrational motor system, small amplitudes trap it within local energy regions. This energetic confinement leads to the emergence of non-ergodicity. Increasing the amplitude provides sufficient energy for the system to overcome potential barriers, enabling a more effective exploration of the entire phase space and thereby achieving ergodicity.

In contrast to the effect of amplitude, an increase in driving frequency drives the system from an ergodic to a non-ergodic state (Figure 3e–h). Higher driving frequencies prevent the system from responding promptly to changes in the external force, analogous to the Non-Poisson Recovery events observed in molecular diffusion [13,23]. In such systems, the memory of previous states is retained over long timescales, leading to a breakdown of ergodicity and a sluggish response to external perturbations. For instance, studies on non-Brownian diffusion in lipid membranes reveal that rapid changes in external conditions hinder the system from reaching a stable ergodic state [20]. Moreover, recent research by Liang et al. indicates that the non-ergodic characteristics of more general stochastic processes utilizing nonlinear clocks have been further examined, highlighting how spatiotemporal changes influence non-ergodicity [55]. In our vibrational motor system, the rapid variations induced by high-frequency external forces similarly impede the ability of the system to achieve ergodicity.

Concerning ageing characteristics, pronounced ageing effects are observed at small amplitudes and high frequencies (Figure 6a,d). This aligns with the ageing behavior in glassy systems, where systems exhibit non-stationary dynamics over long time scales [30,31]. At smaller amplitudes, ageing effects are more prominent. Due to the difficulty the system experiences in escaping local energy traps, its phase space exploration capability diminishes over time, leading to more pronounced ageing features. Conversely, at larger amplitudes and lower frequencies, the system more readily overcomes these traps, and ageing effects are relatively weaker.

As shown in Figure 7, the system exhibits pronounced ageing characteristics at small amplitudes and high frequencies. Specifically, for a small driving amplitude (a=0.1, Figure 7a), Λ decreases with ageing time and eventually stabilizes at Λ=0.02. Under high-frequency driving (ω=5, Figure 7b), Λ fluctuates around unity as the ageing time increases. These persistent deviations from Λ=1 indicate the presence of sustained ageing effects and ergodicity breaking in the system. This is consistent with weak ergodicity-breaking scenarios, where the time average does not converge to the ensemble average, even in the long-time limit [13,29]. Such behavior aligns with Bouchaud’s predictions for disordered systems, where ergodicity remains broken over extended periods due to the system being trapped in a hierarchy of metastable states, resulting in slow relaxation and persistent memory of initial conditions [27]. It is worthy of note that larger driving frequencies induce fluctuations in the ageing behavior (Figure 7b, ω=5). As the frequency increases, the system undergoes more frequent temporal variations under the external force, placing it in a more dynamically evolving state.The ageing factor fluctuates around Λ = 1. This is in agreement with findings reported in studies of the non-equilibrium dynamics of long—range spin—glass models [28,56], where the system response to time—varying external stimuli exhibits explicit time dependence. This time dependence leads to fluctuations in the ageing behavior, consistent with the phenomena observed in the vibrational motor system.

## 6. Conclusions

We report on the ergodicity breaking and ageing of a vibration motor system driven by periodic external forces. The results show that the emergence of non-ergodic behavior stems from two different mechanisms. The system remains localized within the potential well when the driving energy is insufficient at small amplitudes. Under high-frequency driving, the dynamic response is further suppressed because the driving period is shorter than the system’s intrinsic relaxation time. In contrast, increasing the driving amplitude provides sufficient energy for the system to overcome the potential barrier, while reducing the driving frequency allows the system to more effectively follow the external forcing. Furthermore, the ageing properties are most pronounced at low amplitudes and high frequencies, as evidenced by the explicit dependence of dynamical observables on the waiting time, reflecting the breakdown of time-translation invariance. These results reveal how the interaction between amplitude and frequency controls the ergodicity and ageing properties of vibrational motors, providing insights into the operational stability and long-term behavior of vibrational motors.

## Data Availability

Data is contained within the article.
